# NO, ROS, RAS, and PVAT: More Than a Soup of Letters

**DOI:** 10.3389/fphys.2021.640021

**Published:** 2021-02-10

**Authors:** Clarissa Germano Barp, Daniella Bonaventura, Jamil Assreuy

**Affiliations:** ^1^Department of Pharmacology, Centre of Biological Sciences, Universidade Federal de Santa Catarina, Florianópolis, Brazil; ^2^Department of Pharmacology, Institute of Biological Sciences, Universidade Federal de Minas Gerais, Belo Horizonte, Brazil

**Keywords:** perivascular adipose tissue, nitric oxide, vascular dysfunction, angiotensin, superoxide, sepsis

## Abstract

Perivascular adipose tissue (PVAT) has recently entered in the realm of cardiovascular diseases as a putative target for intervention. Notwithstanding its relevance, there is still a long way before the role of PVAT in physiology and pathology is fully understood. The general idea that PVAT anti-contractile effect is beneficial and its pro-contractile effect is harmful is being questioned by several reports. The role of some PVAT important products or systems such as nitric oxide (NO), reactive oxygen species (ROS), and RAS may vary depending on the context, disease, place of production, etc., which adds doubts on how mediators of PVAT anti- and pro-contractile effects are called to action and their final result. This short review will address some points regarding NO, ROS, and RAS in the beneficial and harmful roles of PVAT.

## Introduction

**FIGURE 1:**
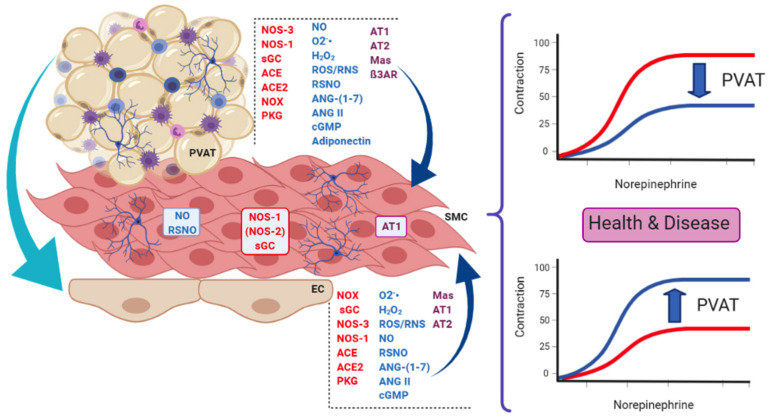
Some of the main actors present in endothelial cells (EC) and perivascular adipose tissue (PVAT) that influence smooth muscle cells (SMC) in the context of the present review. PVAT can also modulate endothelial function and influence the vascular tone. These actors are involved in the dual role of PVAT, namely, its anti-contractile (upper curves) and pro-contractile (lower curves) effects on vessel response, both relevant in health and disease. Enzymes are in red letters, mediators in blue, and receptors/effectors in purple. PVAT contains adipocytes, innervation (neurons) and immune cells (lymphocytes, macrophages, and eosinophils), and vessels (not shown). NOS-3, NOS endothelial isoform; NOS-1, NOS neuronal isoform; (NOS-2), NOS inducible isoform; NOX, NADPH oxidases; sGC, soluble guanylate cyclase; ACE, angiotensin-converting enzyme; ACE2, ACE isoform 2; NO, nitric oxide; O2^-^, superoxide anion; H_2_O_2_, hydrogen peroxide; ROS/RNS, reactive oxygen and nitrogen species, respectively; RSNO, low-molecular weight and protein S-nitrosothiols; ANG-(1–7), angiotensin 1–7; ANG II, angiotensin II; cGMP, cyclic GMP; AT1, angiotensin AT1 receptor; AT2, angiotensin AT2 receptor; Mas, Mas receptor; ß3AR, beta-3 adrenergic receptor; PKG, protein kinase G.

Since the pioneering work of Soltis and Cassis (1991), perivascular adipose tissue (PVAT) has been recognized as an active player in vascular physiology and pathology. Besides adipocytes that are its main cellular component, PVAT also contains fibroblasts, endothelial and immune cells (macrophages, lymphocytes, and eosinophils), extracellular matrix, and adrenergic nerves endings (see Figure 1). Depending on the vessel type, PVAT may have white or brown adipose tissue. In addition, the same vessel may have the two types in different segments (such as the aorta) or even a mixture of both (renal artery, for example; Padilla et al., 2013). Although there are excellent reviews (Ayala-Lopez and Watts, 2017; Fernandez-Alfonso et al., 2017; Saxton et al., 2019) concerning several aspects of PVAT, some components of the vast array of PVAT products have received less attention, namely, the renin–angiotensin system (RAS), nitric oxide (NO), and reactive oxygen species (ROS). This short review will address findings concerning these systems in the beneficial and harmful roles of PVAT.

## PVAT and Renin–Angiotensin System

Renin–angiotensin system has been described as a hormonal system involved in blood pressure regulation and water balance. In this context, the main elements of this system are represented by the angiotensin-converting enzyme (ACE), angiotensin II (ANG II), and AT1 and AT2 receptors (Schiavone et al., 1988; Campagnole-Santos et al., 1989). Later, it has been demonstrated the existence of a counterregulatory RAS axis composed by ACE-2 (Donoghue et al., 2000; Tipnis et al., 2000), the biologically active peptide ANG-(1–7), and Mas receptor (Santos et al., 2003a,b, 2004; Mercure et al., 2008; Santiago et al., 2010).

There are few studies about the function of RAS in PVAT, but it has been shown that it contributes to vascular tone, structural and functional alterations, as well as local inflammatory process (Ferreira et al., 2010). RAS was first described in PVAT when a substantial amount of angiotensinogen mRNA was found in PVAT (Cassis et al., 1988). Later, ACE and ANG II were detected by immunohistochemistry in mesenteric arteries PVAT (Lu et al., 2010). Mas receptor and ANG-(1–7) have also been found in PVAT (Lee et al., 2009; Nobrega et al., 2019). Interestingly, although all RAS components, except renin, were expressed in both adipose tissue types present in PVAT, the expression of renin/prorenin receptor, angiotensinogen, and AT1 and AT2 receptor was higher in PVAT containing white adipose tissue (Gálvez-Prieto et al., 2008).

The involvement of RAS from PVAT on vascular tone is dual. The pro-contractile effect was observed in mesenteric arteries stimulated by electrical field, where PVAT generated ANG II, enhancing the contraction (Lu et al., 2010). In non-physiological situations, such as hypoxia, PVAT from small arteries lost its anti-contractile activity due to PVAT-mediated ANG II secretion (Greenstein et al., 2009; Rosei et al., 2015). In the same line, PVAT from thoracic aorta of rats subjected to myocardial infarction-induced heart failure becomes dysfunctional due to overactivation of RAS characterized by increased activity/levels of ACE1, angiotensin II, and AT1 and AT2 receptors, thus enhancing oxidative stress and, consequently, reducing NO bioavailability (Fontes et al., 2020). On the other hand, studies performed in large arteries and veins showed that ANG-(1–7) is one of the PVAT-derived relaxing factors (PVDRFs). For example, ANG-(1–7) levels released by PVAT were reduced in hypertensive rats (SHR model) (Lu et al., 2011a). As for the mechanisms involved in PVAT RAS anti-contractile effects, ANG-(1–7) released by PVAT acts by Mas receptor on endothelial cells, releasing NO and leading to relaxation of the blood vessels (Lee et al., 2009, 2011; Lu et al., 2011b). These findings have been expanded by showing that aorta associated PVAT displays Mas and AT2 receptors, PI3k/Akt pathway, as well as endothelial (eNOS; NOS-3) and neuronal (nNOS; NOS-1) NO synthase (NOS) isoforms. The presence of these receptors and enzymes in PVAT is responsible for NO and hydrogen peroxide production due to NOS-3 and NOS-1 isoforms’ activation, respectively (Nobrega et al., 2019).

Besides directly affecting vessel tonus, RAS is also involved in PVAT inflammatory and immune actions on vessel function. For example, PVAT releases adipokines, such as adiponectin (Chatterjee et al., 2009) that plays a cardiovascular protection role which is related to its anti-inflammatory (Matsuzawa, 2006) and anti-proliferative effects (Wang et al., 2005). Treatment of hypertensive patients with ACE inhibitor or AT1 receptor antagonist increased adiponectin concentrations (Furuhashi et al., 2003), suggesting that PVAT RAS may indeed have a proinflammatory effect. Furthermore, when perivascular fat is submitted to proinflammatory insults, such as aldosterone and hypoxia, macrophages are activated leading to the loss of anti-contractile activity of healthy PVAT (Greenstein et al., 2009; Withers et al., 2011). Moreover, AT1 receptors from PVAT shift macrophage to proinflammatory (M1) polarization and this effect is involved in local inflammation and matrix metalloproteinase (MMP) activation, contributing to the pathological environment, such as for aneurysm formation (Sakaue et al., 2017).

In summary, PVAT RAS is involved in vascular tonus maintenance in physiological and pathophysiological situations, as well as in the inflammatory and immune aspects affecting vessel function.

## PVAT and NO/ROS

Perivascular adipose tissue adipocytes express NOS-3, but the enzyme is also expressed in PVAT endothelial cells (Ribiere et al., 1996; Araujo et al., 2011; Xia et al., 2016). NOS-1 has also been found in PVAT (Nobrega et al., 2019). The expression of PVAT NOS-3 seems to be variable between vessels and it can vary even in the same vessel (Victorio et al., 2016). NOS-3 from PVAT shares several characteristics with the endothelial isoform such as the need of dimerization for catalysis, chaperone dependency, L-arginine concentrations, and the need of BH4 (Victorio and Davel, 2020). NOS enzymes and in particular PVAT NOS-3 produce superoxide anion when uncoupled (Vasquez-Vivar et al., 2003; Margaritis et al., 2013; Nobrega et al., 2019). Although several conditions are known to reduce NOS-3 activity in PVAT (lack of L-arginine, reduction in serine 1177 phosphorylation and acetylation; Man et al., 2020), BH4 oxidation and its reduced availability probably are the main cause for NOS-3 uncoupling in endothelial cells and most likely in PVAT as well (Vasquez-Vivar et al., 2003; Marchesi et al., 2009). Since not all NOS molecules are uncoupled at the same time, some of them produce NO while some produce superoxide anion. Superoxide anion and NO can react stoichiometrically to produce peroxynitrite, a powerful oxidant (reviewed in Beckman, 2009). Adiponectin, one of the main PVAT products, can recouple NOS-3, improve redox state *via* PI3/Akt-mediated phosphorylation of NOS-3, and increase BH4 bioavailability (Margaritis et al., 2013).

NO produced by PVAT indeed has important physiological and pathological effects and inhibition or increase in its production affects vessel response (see, for example, the excellent reviews by Zaborska et al., 2017; Daiber et al., 2019; Nava and Llorens, 2019). NO produced in PVAT uses canonical pathways to evoke its effect on vessels such as calcium-dependent potassium channels (Gao et al., 2007; Lee et al., 2009) and cyclic GMP-dependent protein kinase (PKG) (Withers et al., 2014). PVAT adipocytes also express beta-3 adrenergic receptors and its stimulation increases cAMP levels, activates voltage-dependent potassium channels isoform 7 (Kv7), and induces NO release (Bussey et al., 2018). However, PVAT also releases a transferable relaxing factor that acts by tyrosine kinase-dependent activation of potassium channels that are not NO (Lohn et al., 2002).

Although NOS uncoupling is an important ROS producer, PVAT also generates ROS from other sources such as mitochondria and NOX (Gao et al., 2006; Nosalski and Guzik, 2017). Mitochondrial contribution comes mainly from electron transport chain by generating superoxide anions (Costa et al., 2016). Whereas small amounts of ROS are important for inter and intracellular signals for cell physiology, higher amounts of them can be detrimental (Garcia-Redondo et al., 2016; Krylatov et al., 2018). Notwithstanding the importance of superoxide, its short half-life and radius of diffusion (Fridovich, 1995) make its stable metabolite hydrogen peroxide more relevant for direct ROS effects in PVAT (Ardanaz and Pagano, 2006; Gao et al., 2007). Hydrogen peroxide can be formed by superoxide dismutation carried out by SOD or, particularly relevant for PVAT containing brown adipose tissue, by the activity of NOX4 which produces hydrogen peroxide directly (Friederich-Persson et al., 2017).

Whereas the effect and role of NO in PVAT anti-contractile effect are relatively well established, the effects of ROS produced by PVAT are still unsettled. For instance, it is widely accepted that superoxide anion produced in excess may reduce NO bioavailability, thus decreasing PVAT anti-contractile effect (Marchesi et al., 2009). However, ROS may directly act as mediators of PVAT anti-contractile effects (Costa et al., 2016; Chang et al., 2020). For example, electrical field stimulation and perivascular nerve activation enhance PVAT pro-contractile effect due to superoxide release and MAPK/ERK activation (Gao et al., 2006). Hydrogen peroxide in turn relaxes vessels *via* potassium channel activation and/or *via* soluble guanylate cyclase/PKG activation (Gao et al., 2007; Withers et al., 2014; Friederich-Persson et al., 2017). Moreover, specific ROS can originate opposing effects depending on its local of production. For instance, hydrogen peroxide produced by the vessel induces vasoconstriction in hypertension and cardiovascular disease, but the same species exhibit vasorelaxant properties when produced by PVAT (Ardanaz and Pagano, 2006; Gao et al., 2007).

Perivascular adipose tissue from mouse thoracic aorta expresses Mas and AT2 receptors as well as NOS-3 and NOS-1, being the products NO and hydrogen peroxide relevant effectors for PVAT anti-contractile effect toward phenylephrine. Interestingly, this PVAT anti-contractile effect is only verified in the absence of vascular endothelium (Nobrega et al., 2019). Since it has been suggested that PVAT has both endothelium-dependent and -independent pathways affecting vascular tone (Gao et al., 2007), collectively these findings raise important questions on the relevance of endothelium to PVAT effects, what is the role of PVAT in endothelial dysfunction and if this interaction is the same in different species.

In summary, the role of NO and ROS (and their potential interaction) in PVAT pathological role still needs more studies since antioxidant approaches as therapeutic options have failed to improve PVAT actions in cardiovascular diseases.

## Beneficial Effects of PVAT

In physiological situations, PVAT has a global anti-contractile effect that can be seen in different vessels such as the aorta and mesentery arteries, as well as in veins. These PVAT anti-contractile effects are thought to contribute to the vascular tonus maintenance (Soltis and Cassis, 1991; Gao et al., 2005; Lu et al., 2011b). However, PVAT influence on vessel tonus may vary depending on its adipose tissue composition and/or vessel location. For instance, in the thoracic aorta, PVAT adipose tissue is composed by brown adipose tissue (BAT), whereas in the abdominal aorta and mesenteric arteries, it is mostly composed by white adipose tissue (WAT; Padilla et al., 2013; Brown et al., 2014). This difference does not concern only to the phenotype, but also to PVAT paracrine actions, since the influence of PVAT on contractility of the abdominal aorta is smaller compared to thoracic aorta. The different quantitative anti-contractile effect is thought to be related to a lower expression of NOS-3 in the abdominal segment (Victorio et al., 2016).

The activation of endothelial Mas receptor by ANG-(1–7) released by PVAT has also been found to be a relevant anti-contractile mechanism in the aorta (Lee et al., 2011). NO acts as a key downstream effector not only for ANG-(1–7), but it is relevant for the anti-contractile effect of PVAT-derived adipokines as well, in both arterial and venous vascular networks. For example, endothelium-dependent dilation induced by adiponectin (Chen et al., 2003; Lynch et al., 2013) and leptin (Vecchione et al., 2002; Gálvez-Prieto et al., 2012) also relies, at least in part, on the NO production through phosphorylation and activation of NOS-3.

Perivascular adipose tissue surrounding veins also affects their tonus *via* NO and ROS production (Lu et al., 2011b). For example, in rat-isolated vena cava, PVAT reduced the contraction elicited by phenylephrine, serotonin, and thromboxane-A2 mimetic vasoconstrictor (U46619), indicating that PVAT anti-contractile effect is agonist independent. Interestingly, the contraction in vena cava is only attenuated by PVAT when in the presence of endothelium and this effect is related to Mas receptors activation and subsequent NO production (Lu et al., 2011b).

Besides being an important player in physiological situations, PVAT can show beneficial actions in cardiovascular diseases, such as atherosclerosis. For instance, PVAT displays an endothelial protective effect in the LDLr-KO model of atherosclerosis through the compensatory increase in PVAT NOS-3 expression (in sharp contrast to the lack of NOS-3 expression in the endothelium), thus leading to the recovery of acetylcholine relaxation in the early stages of atherosclerosis development (Baltieri et al., 2018). Another PVAT protective effect relates to the increase in adiponectin levels leading to prevention of plaque formation by recoupling NOS-3 and increasing NO bioavailability, and also through macrophage autophagy induction *via* suppression of Akt/FOXO3 (Margaritis et al., 2013; Li et al., 2015). Of note, increases in adiponectin gene expression can be induced by vascular superoxide and by products of lipid peroxidation, thus representing a local mechanism to control oxidative stress (Margaritis et al., 2013). Furthermore, Mas receptor activation with the agonist AVE0991 induced anti-atherosclerotic and anti-inflammatory actions by reducing monocyte/macrophage differentiation and recruitment to PVAT during early stages of atherosclerosis in ApoE−/− mice (Skiba et al., 2017).

## Harmful Effects of PVAT

Akin to the endothelial dysfunction, PVAT dysfunction can be an early marker of vascular disease. Experiments in aorta rings from pre-hypertensive SHR rats have shown that the reduction in the PVAT anti-contractile effect precedes the establishment of hypertension (Gálvez-Prieto et al., 2008). As for the mechanisms involved, the lack of PVAT anti-contractile response during hypertension is caused, at least in part, by a reduction in leptin production and impaired activation of NOS-3 (Gálvez-Prieto et al., 2012). The anti-contractile effect of PVAT is completely abolished in models of obesity induced by high-fat diet (HFD) and the New Zealand genetic model (NZO), and significantly reduced in the ob/ob genetic model (Marchesi et al., 2009; Ketonen et al., 2010; Agabiti-Rosei et al., 2014). Animals treated with HFD showed uncoupling of NOS-3 and decreased availability of its substrate arginine, leading to a reduction in NO production and increasing superoxide anion production, well known for its pro-contractile action (Gil-Ortega et al., 2014; Xia et al., 2016). In addition to NO and ROS, other local changes are known to shift the PVAT effect in obesity, including changes in the size and mass of the adipocytes, changes in the secretory profile of the PVAT adipose tissue, and the reduction in density and formation of capillaries, providing a hypoxic environment (Marchesi et al., 2009). Hypoxia, in turn, increases the production and release of proinflammatory cytokines, chemokines (MIP-1α and MCP-1), and leptin, inducing the infiltration of immune cells and decreasing local adiponectin production with consequent downregulation of NOS-3, thus favoring endothelial dysfunction (Chatterjee et al., 2009; Greenstein et al., 2009; Ketonen et al., 2010). As stated above, immune cells, such as macrophages, have an important influence on PVAT dysfunction, as they induce NOX and NOS-2 (iNOS; inducible isoform) activity, resulting in an increased production of superoxide anion and other ROS, such as peroxynitrite. The local increase of NOX goes along with the increased expression of its p67phox subunit in the PVAT of obese animals (Ketonen et al., 2010; DeVallance et al., 2018). This communication between hypoxia-inflammation-oxidative stress leads to proinflammatory cytokine and adipokine production, such as leptin, TNF-α, and IL-6 and the negative regulation of anti-inflammatory mediators, such as adiponectin and IL-10, thus aggravating endothelial dysfunction, resulting in the loss or attenuation of the anti-contractile action of PVAT in obesity (Xia and Li, 2017).

In addition to hypertension and obesity, a shift in PVAT genes and proteins content/expression associated with oxidative damage and inflammation in experimental models of atherosclerosis has been observed. This local inflammatory profile includes increases in MCP-1, IL-6, and angiopoietin-like protein 2 (Angptl2), supporting immune cell migration and accelerating neointima hyperplasia (Viedt et al., 2002; Tian et al., 2013; Manka et al., 2014; Quesada et al., 2018). Moreover, both components of RAS and macrophage markers were upregulated in PVAT in ApoE−/− mice fed with a high-cholesterol diet. PVAT transplantation from these animals into ApoE−/− recipient mice fed a normal chow diet induced an increase in atherosclerosis development. This effect was significantly reduced by blocking ANG II receptors or by transplanting PVAT from mice lacking AT1 receptors, pointing to a RAS-dependent mechanism of PVAT inflammation during atherosclerosis (Irie et al., 2015).

A hitherto unknown involvement of PVAT in vascular dysfunction of septic shock has been recently addressed. Sepsis vascular dysfunction is characterized by the loss of response to vasoconstrictors and profound hypotension. Paradoxically, in this pathological condition, the anti-contractile effect of PVAT is increased. *Ex vivo* experiments using aorta rings with intact PVAT showed a worsening in the response to norepinephrine, phenylephrine, and serotonin (Awata et al., 2019; Barp et al., 2020). The increased PVAT anti-contractile effect in sepsis could be entirely attributed to PVAT, since PVAT was taken from septic aorta and mesenteric artery and then incubated with healthy vessel rings stripped of their own PVAT reduced norepinephrine effects (Barp et al., 2020). The mechanisms seem to be dependent on the PVAT location/phenotype. In septic thoracic aorta PVAT (brown adipose tissue phenotype), the mechanism is related to an increase in beta-3 adrenergic receptor (known to induce adiponectin and to increase NO production) density and also in NOS-1 expression, leading to an increase in NO production which acts through soluble guanylate cyclase and S-nitrosylation (Awata et al., 2019; Barp et al., 2020). However, in superior mesentery (white adipose tissue phenotype), ROS production probably from mitochondrial dysfunction seems to be the main mechanism of vascular dysfunction in this vessel type during sepsis progression (Barp et al., 2020). These reports shed light on the fact that even the accepted beneficial anti-contractile effect of PVAT may turn against the host, evidencing that PVAT contribution to vascular (dys)function is far more complicated.

The communication between the PVAT and the underlying vasculature occurs bidirectionally, and both NO/ROS and RAS systems contribute to vascular homeostasis and to different cardiovascular diseases. However, confirmation of PVAT as a potential target to improve vascular function still demands more studies to understand the mechanisms involved and then enabling new therapeutic approaches.

## Discussion

During last decades, important knowledge about the structure and function of PVAT in cardiovascular maintenance and disease was achieved but there are still many unanswered points such as:

(a) What are the most relevant mechanisms of crosstalk between PVAT and the endothelium?
(b) What are and where are the sensor mechanisms that trigger PVAT anti-contractile and pro-contractile effects?
(c) What is the contribution of NO, ROS, and RAS to the divergent roles of PVAT (e.g., pro- and anti-contractile)?

The real beneficial and harmful effects of PVAT in the vascular systems are far from being fully comprehended. A short compilation (Table 1) taken only from works cited here clearly shows that the variety of protocols, vessel types, stimulus, and species make difficult to see a clear pattern of PVAT influences in vascular physiology and pathology. In particular, the “dual” role of PVAT cannot be simply ascribed to its anti- and pro-contractile effects as each of these effects can be called upon in both physiological and pathological situations. However, and notwithstanding the lack of several crucial information on the mechanisms, PVAT is a putative target for the treatment of cardiovascular diseases.

**TABLE 1:** Type of vessel containing PVAT, observed effect, type of stimulus, source of vessels and/or PVAT, and condition or model in which the study was conducted.

References	Vessel(s)	Effect	Stimulus	Species	Condition/model
Agabiti-Rosei et al., 2014	MA	AC	NE	Mouse	Obesity
Awata et al., 2019	TA	AC	Phe; 5-HT	Rat	Sepsis
Baltieri et al., 2018	TA	AC	ACh; insulin	Mouse	Hypercholesterolemia
Barp et al., 2020	TA; MA	AC	NE	Rat	Sepsis
Bussey et al., 2018	MA	AC	NE	Rat	Physiological
Costa et al., 2016	TA	AC	Phe	Rat	Physiological
DeVallance et al., 2018	TA	PC	ACh; Phe	Rat	Metabolic syndrome
Fontes et al., 2020	TA	PC	Phe	Rat	Heart failure
Friederich-Persson et al., 2017	MA	AC	NE	Mouse	Physiological
Gálvez-Prieto et al., 2012	Aorta	AC	AII	Rat	Hypertension
Gao et al., 2005	TA	AC	U4; Phe	Human	Coronary artery disease
Gao et al., 2006	MA	PC	EFS	Rat	Physiological
Gao et al., 2007	Aorta	AC	Phe; 5-HT	Rat	Physiological
Gil-Ortega et al., 2014	MA	AC	NE	Mouse	Obesity
Greenstein et al., 2009	Sm. arteries	AC	NE	Human	Obesity
Greenstein et al., 2009	MA	AC	NE	Rat	Obesity
Ketonen et al., 2010	Abd. Aorta	PC	Phe	Mouse	Obesity
Lee et al., 2009	Aorta	AC	Phe	Rat	Physiological
Lee et al., 2011	TA	AC	Phe	Mouse	Physiological
Lohn et al., 2002	Aorta	AC	Phe; AII; 5-HT	Rat	Physiological
Lu et al., 2010	MA	PC	EFS; AII	Rat	Physiological
Lu et al., 2011a	Aorta	AC	Phe	Rat	Hypertension
Lu et al., 2011b	IVC	AC	Phe; U4; 5-HT	Rat	Physiological
Lynch et al., 2013	MA	AC	NE	Mouse	Physiological
Marchesi et al., 2009	MA	AC	NE	Mouse	Metabolic syndrome
Margaritis et al., 2013	SV; IMA	AC	ACh; SNP	Human	Coronary artery disease
Nobrega et al., 2019	TA	AC	Phe	Mouse	Physiological
Rosei et al., 2015	MA	AC	NE	Rat	Hypoxia
Soltis and Cassis, 1991	TA	AC	NE	Rat	Physiological
Victorio et al., 2016	TA	AC	Phe	Rat	Physiological
Withers et al., 2011	MA	AC	NE	Mouse	Inflammation
Withers et al., 2014	MA	AC	Phe	Mouse	Physiological/hypoxia

*MA, mesenteric artery; TA, thoracic aorta or thoracic arteries, in human case; Sm. Arteries, small arteries; Abd. Aorta, abdominal aorta; IVC, inferior vena cava; SV, saphenous vein; IMA, internal mammary artery; AC, anti-contractile; PC, pro-contractile; NE, norepinephrine; Phe, phenylephrine; 5-HT, serotonin; Ach, acetylcholine; AII, angiotensin II; EFS, electrical field stimulation; SNP, sodium nitroprusside; U4, U46619.*

## Author Contributions

CB, DB, and JA wrote the manuscript. All authors equally contributed to the article and approved the submitted version.

## Conflict of Interest

The authors declare that the research was conducted in the absence of any commercial or financial relationships that could be construed as a potential conflict of interest.
